# Fatal Dengue Fever in a Traveler Returning from Togo to Germany

**DOI:** 10.4269/ajtmh.25-0203

**Published:** 2026-02-10

**Authors:** Matin Kohsar, Markus Haar, Jonas Schmidt-Chanasit, Michael Ramharter, Bettina M. Buchholz, Susanne Krasemann, Christian Bernreuther, Daniel Cadar, Till Frederick Omansen, Dominic Wichmann, Lina-Hanne Maria Ko, Sabine Jordan

**Affiliations:** ^1^ I. Department of Medicine, Section Tropical Medicine, University Medical Center Hamburg–Eppendorf, Hamburg, Germany;; ^2^ Center for Tropical Medicine, Clinical Research Department, Bernhard-Nocht-Institute for Tropical Medicine, Hamburg, Germany;; ^3^ Center for Anesthesiology and Intensive Care Medicine, Department of Intensive Care Medicine, University Medical Center Hamburg–Eppendorf, Hamburg, Germany;; ^4^ Department of Arbovirology and Entomology, Bernhard-Nocht-Institute for Tropical Medicine, Hamburg, Germany;; ^5^ German Center for Infection Research, Partner Site Hamburg-Lübeck-Borstel-Riems, Hamburg, Germany;; ^6^ Department of Visceral Transplantation, University Medical Center Hamburg–Eppendorf, Hamburg, Germany;; ^7^ Institute of Neuropathology, University Medical Center Hamburg–Eppendorf, Hamburg, Germany;; ^8^ Core Facility for Experimental Histo-Pathology, University Medical Center Hamburg–Eppendorf, Hamburg, Germany;; ^9^ Institute of Pathology, University Medical Center Hamburg–Eppendorf, Hamburg, Germany;; ^10^ Department of Intensive Care Medicine, University Medical Center Hamburg–Eppendorf, Hamburg, Germany

## Abstract

A previously healthy traveler of Togolese origin visiting friends and relatives presented with severe dengue complicated by acute liver failure. Despite intensive care management and listing for high-urgency liver transplantation, the patient succumbed to the disease. This case highlights the risk for life-threatening travel-related complications of dengue.

## INTRODUCTION

Dengue is the most common viral hemorrhagic fever worldwide. The dengue virus (DENV) is mainly transmitted by *Aedes aegypti*, but *Aedes albopictus* may also serve as an efficient vector. During 2023, more than 5 million cases occurred in five WHO regions.^1^ Travelers to highly endemic regions visiting friends and relatives (VFR) are at particular risk of infection. Here, we report a fatal case of acute liver failure owing to severe dengue in a traveler VFR returning from travel to Togo.

## CASE PRESENTATION

A 47-year-old male patient of Togolese origin presented to a hospital in northern Germany during the summer of 2022 complaining of fever, abdominal pain, and exhaustion for 4 days. He returned from a 4-week family visit to Lomé in Togo the same day. During travel, he had contact with a nephew who died of an acute icteric illness of unclear origin. Pre-existing bronchial asthma, mild arterial hypertension, and mild obesity (body mass index of 28) were reported. The patient was vaccinated against yellow fever but did not take chemoprophylaxis for malaria.

Upon initial admission, laboratory results showed thrombocytopenia, cholestatic hepatitis with impaired hepatic function, and acute kidney injury. Intravenous artesunate (2.4 mg/kg) was commenced as malaria could not be ruled out conclusively by microscopy. Serum samples were tested, and they were negative for HIV-1 antibodies and antigen, hepatitis B surface antigen, and hepatitis C virus antibodies.

The following day, the patient was transferred to the intensive care unit of our tertiary referral center with acute liver failure, lactic acidosis, and elevated inflammatory markers. On referral, the patient was hemodynamically stable, conscious, and alert. Abdominal pain was present and predominantly located in the upper-right quadrant. Abdominal computed tomography revealed no infectious focus. Hepatic and portal veins were patent, the liver was enlarged, and the gallbladder wall was thickened. Surgical consultation ruled out acute cholecystitis.

Broad-spectrum anti-infective therapy with ceftriaxone and metronidazole was started as typhoid fever with secondary liver failure owing to septic shock was among the initial differential diagnoses. Malaria was ruled out by microscopy and rapid test, and blood cultures did not reveal bacterial bloodstream infection. Samples were tested for hemorrhagic fever viruses, including dengue, Rift Valley fever, *Phlebovirus* spp., hantavirus, filoviruses, Lassa virus, and yellow fever virus, at the national reference center for tropical infections at the Bernhard Nocht Institute for Tropical Medicine.

Dengue NS1 rapid test, DENV reverse transcription polymerase chain reaction (cycle threshold 32), and anti-DENV IgG (titer of 1:5,120) came back positive during the afternoon of referral to our center, whereas anti-DENV IgM was negative, suggesting an acute secondary DENV infection. Other pathogens, namely *Rickettsia* spp., *Brucella* spp., *Coxiella* spp., *Leptospira* spp., *Plasmodium* spp., *Schistosoma* spp., hepatitis E virus, hepatitis A virus, hepatitis B virus, hepatitis C virus, cytomegalovirus, Epstein–Barr virus, and HIV, were tested, and the results were negative.

The patient’s clinical condition deteriorated rapidly during the first day of referral with progressive somnolence because of hepatic encephalopathy. *N*-acetylcystein, rifaximin, L-ornithinaspartate, and lactulose therapy were administered, and the patient was listed for high-urgency liver transplantation given that rapid assessment with cranial computed tomography, echocardiography, and psychological consultation with the next of kin did not reveal any contraindications for transplantation. The next day, continuous renal replacement therapy was commenced because of anuria, hyperkalemia, and lactic acidosis. The patient was intubated based on diminished protective airway reflexes and severe acute respiratory distress syndrome.

Lactic acid levels were persistently high as was the need for inotropes, which escalated further during the third day of intensive care management (Supplemental Figure 1). Severe coagulopathy was corrected by fibrinogen, coagulation factors, and thrombocyte replacement. Systemic capillary leakage caused massive fluid retention and consequently, abdominal compartment syndrome. An emergency laparotomy was performed to relieve the intra-abdominal pressure. The liver parenchyma appeared pale and stiff, consistent with acute liver failure. The open abdomen limited the use of the prone position to treat severe acute respiratory distress syndrome.

As the patient further deteriorated regarding liver function tests and lactate dehydrogenase, toxic liver syndrome with massive hepatocellular necrosis and cytokine release was suspected. Salvage recipient hepatectomy with placement of a temporary portocaval shunt was considered but ultimately not performed given the unpredictable time to compatible donor organ availability.^2^ To further bridge the time to transplant and limit the cytokine storm, two continuous venovenous hemodiafiltration machines with cytokine adsorbers were set up.

We received a single organ offer during the night of the third day; however, the organ was not transplantable based on poor quality. Severe cardiopulmonary failure was nonresponsive to all measures taken, including inhaled nitric oxide therapy, and it placed our patient at risk to be unfit for liver transplantation. Lacking alternative management options, salvage recipient hepatectomy was reconsidered on the fourth day along with consecutive intraoperative establishment of venovenous extracorporeal membrane oxygenation therapy. This ultimate decision was only made after extensive interdisciplinary case discussion and in the hope that an acceptable donor organ would become available within the next 24–36 hours. Unfortunately, the patient became asystolic before the intervention and died despite prolonged resuscitation measures.

**Figure 1.**
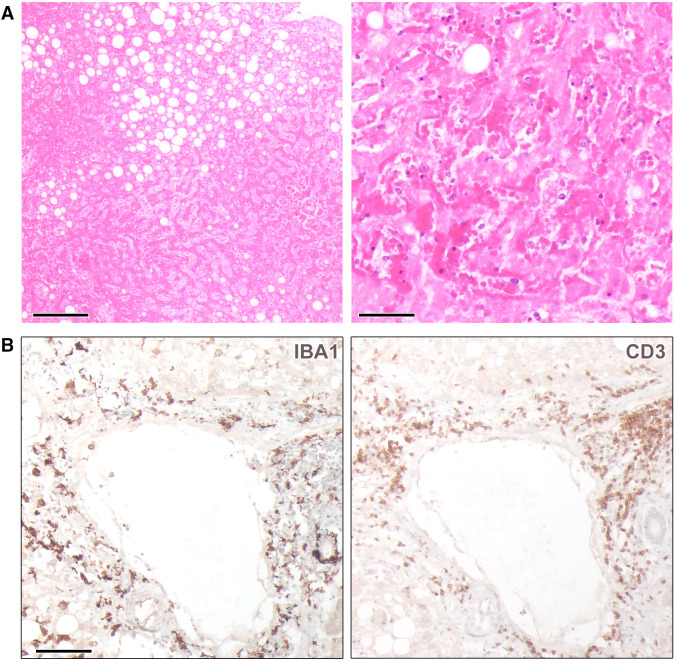
Widespread hepatocellular necrosis with activation of immune cells in liver tissue. (**A**) Representative hematoxylin and eosin staining of the patient’s liver tissue displays widespread necrosis comprising 90% of the parenchyma. The tissue overview shows moderate steatosis (30% macrovesicular and 10% microvesicular), which is likely related to pre-existing metabolic dysfunction-associated steatotic liver disease (left panel). No evidence of thrombotic events, excessive bleeding, malignancy, fibrotic changes, or liver cirrhosis could be detected. Scale bar: 200 *µ*m. The close-up view in the right panel reveals almost complete destruction of liver tissue by necrosis. Scale bar: 50 *µ*m. (**B**) Immunohistochemical staining of Kupffer cells/macrophages/monocytes (IBA1) shows highly activated cell morphology with increased numbers in liver parenchyma and around vessels. Localization of IBA1^+^ cells within the vessel structure and bile ducts is indicative for loss of vascular and functional integrity. Higher densities of T cells (CD3) are mainly found clustered around blood vessels. Scale bar: 100 *µ*m.

Postmortem histological evaluation revealed hepatocellular necrosis of about 90% of the patient’s liver parenchyma and perivascular immune cell activation, in line with hematogenous viral spread (Figure 1). Viral metagenomic sequencing of the patient’s liver sample was performed using a NextSeq (Illumina, San Diego, CA) 2000 sequencing platform as described by Cadar et al.^3^ Sequence analysis and phylogenetic investigation identified the presence of DENV type 3, specifically the major and minor lineage B.2 (Supplemental Figure 2) that predominantly circulates in West Africa. This classification aligns with the recently proposed DENV lineage nomenclature (https://dengue-lineages.org/).

## DISCUSSION

Acute liver failure is a rare complication of dengue among adult patients.^4^ Data on the incidence of acute liver failure secondary to dengue are rather scarce and mostly limited to Southeast Asia (namely Thailand and India), where studies report incidence rates among hospitalized patients of 0.3–1.1% with high mortality rates of around 60%.^5^ Retrospective studies from India report dengue as a common cause of acute liver failure among pediatric patients.^6^^,^^7^

There is currently no licensed DENV-specific antiviral therapy.^4^ Although a randomized trial showed no association with adverse effects in early corticosteroid treatment, there is no evidence for its efficacy in dengue.^8^ Liver transplantation has rarely been performed in DENV-induced acute liver failure (e.g., in 2019 and 2021).^9^^,^^10^ Our case reported here highlights the difficulties to manage persistent dengue shock syndrome and the failure of conservative management. Liver transplantation could have been the most valuable therapeutic option assuming that the pathogenesis was predominantly driven by cytokine storm and massive hepatocellular necrosis rather than by viral replication. However, this case also points out the real-life barriers to lifesaving liver transplantation, namely donor organ shortage and transplant candidate death on the waiting list because of multiorgan failure. Another noteworthy aspect is the gall bladder wall thickening—most likely caused by systemic capillary leakage and edema—that could be mistaken as acute cholecystitis as reported in a fatal case of dengue hemorrhagic fever in Germany.^11^

With Qdenga^®^ (Takeda, Tokyo, Japan) being approved by the European Commission, there is now a vaccine available for travelers to areas with high risk of dengue transmission.^12^^–^^14^ It is licensed for individuals older than 4 years old irrespective of their dengue serostatus.^15^ However, in many European countries, including Germany, Qdenga is primarily recommended for seropositive travelers. The case presented here highlights that previous infections may be unknown, and vaccination should be considered in travelers VFR from dengue-endemic regions with high probability of seropositivity. The fact that the patient also did not take malaria chemoprophylaxis suggests that no prior travel health consultation was sought, which is a common problem that we encounter in our travel medicine clinic. Still, travel medicine consultations often lack specific engagement strategies for travelers VFR who often need more specialized travel advise because of different risk behaviors from common tourists, to whom most travel health services are tailored. Individual risk factors regarding predisposing medical conditions and travel mode should be discussed in this regard as well.

## CONCLUSION

The case presented here highlights a rare but highly significant complication of dengue. Dengue-associated acute liver failure is difficult to manage even in high-resource settings, and the expected surge of dengue transmission will pose new challenges on clinical management of cases and travel medicine services.

## Supplemental Materials

10.4269/ajtmh.25-0203 Supplemental Materials
