# Supplementary material for: Fatal Dengue Fever in a Traveler Returning from Togo to Germany
Source: Am J Trop Med Hyg. 2026 Feb 10;114(4):720–3. doi: 10.4269/ajtmh.25-0203 (PMC13045631; doi:10.4269/ajtmh.25-0203)
Supplement: Supplemental Materials [file tpmd250203.SD1.pdf]

Supplemental Figure 1. Overview of the patient´s hepatic (upper panel), hemodynamic (center), and coagulation (lower panel) parameters during treatment at our intensive care unit. Data are shown over time from referral to our center (0 hours) until death on the fourth day (hour 79).

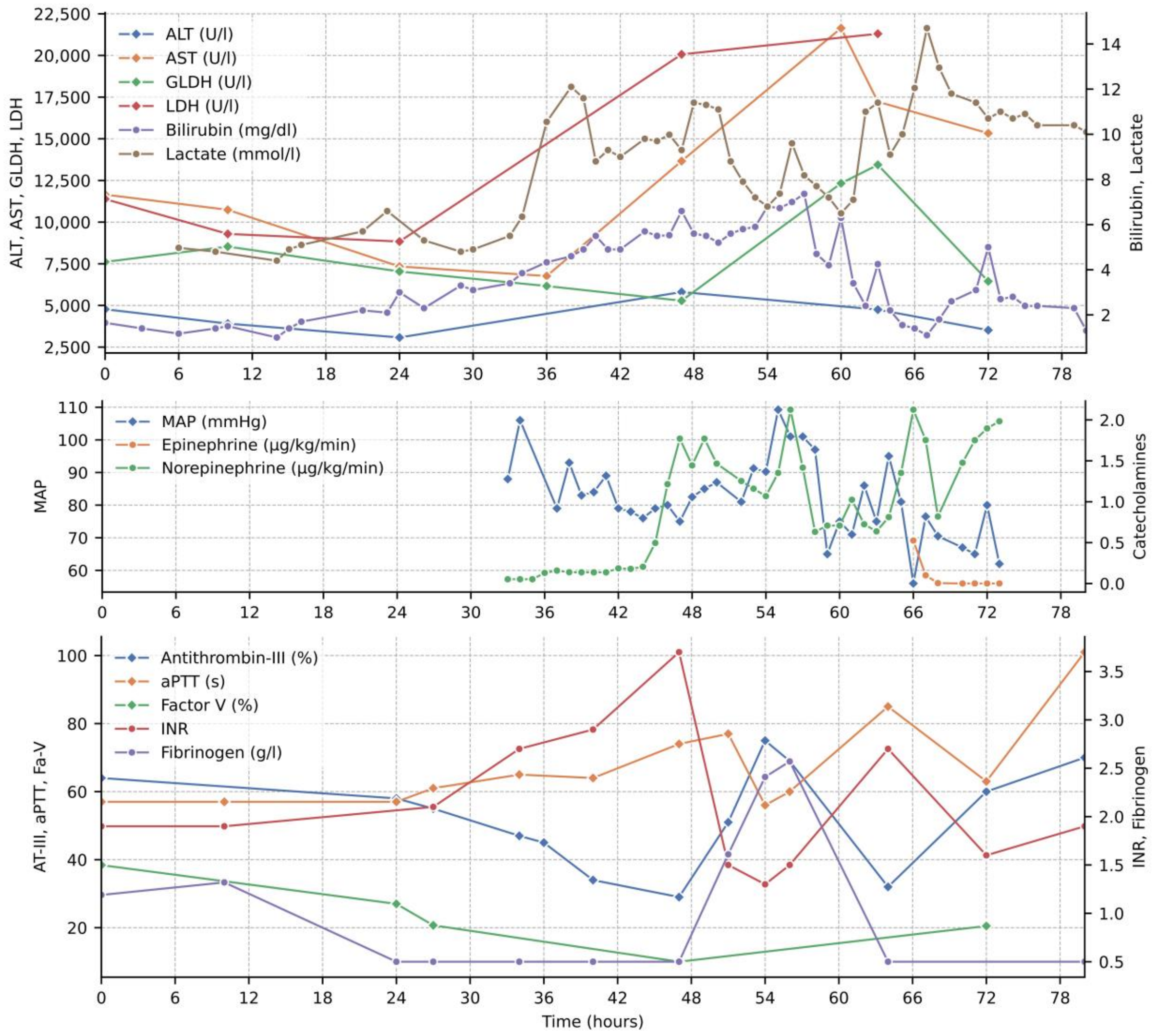

Supplemental Figure 2. Phylogenetic relationships based on complete envelope gene sequences of the dengue virus (DENV) strain isolated from the patient's liver via metagenomic next-generation sequencing (mNGS). The genotype and major/minor lineage grouping of our sequence, along with the geographic origins and year of isolation of the lineage members, are indicated. Node circles represent the bootstrap values as shown in the legend. The scale bar represents nucleotide substitutions per site.

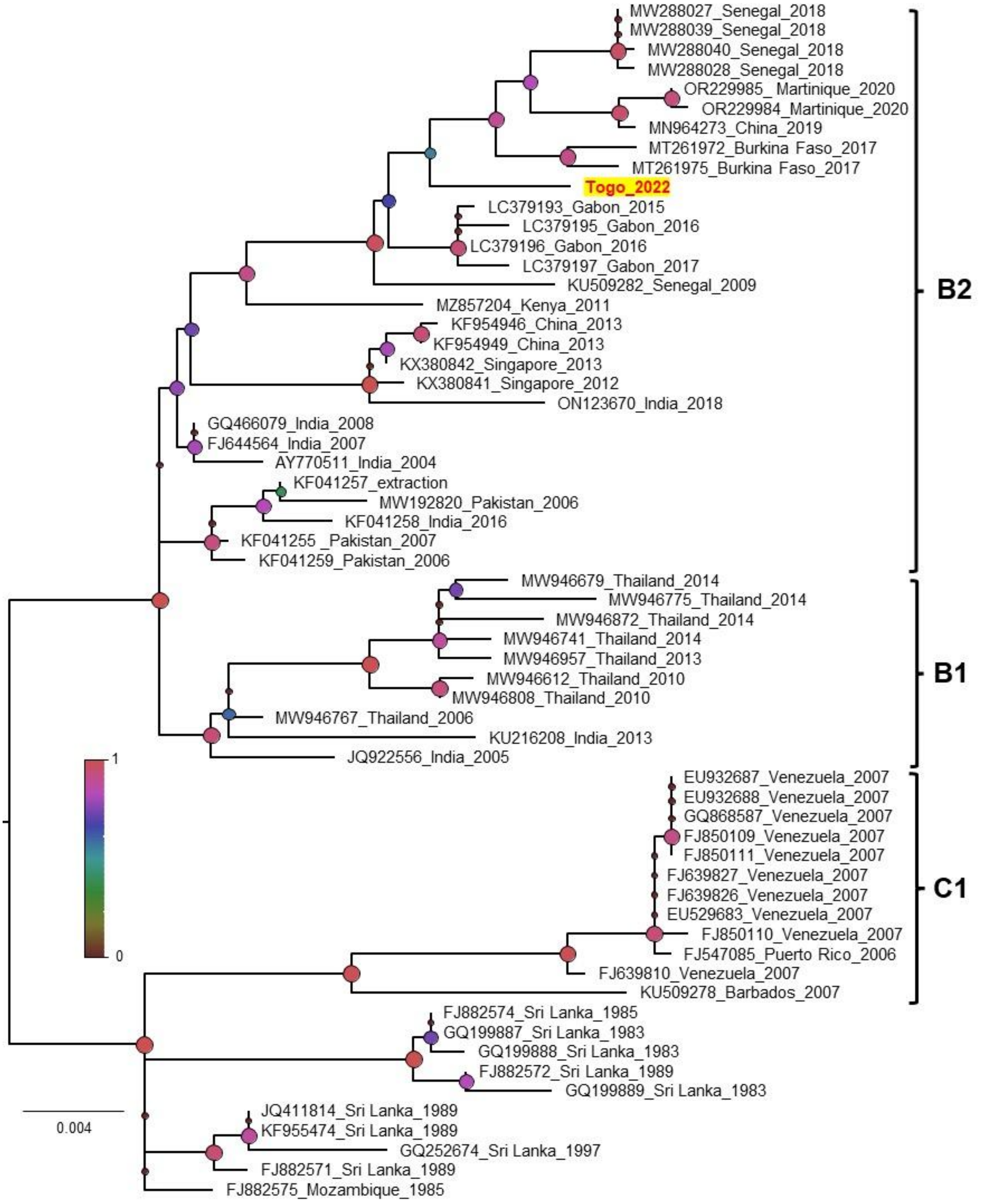

Supplementary data

Supplemental data 1

Histopathological Method for Figure 1:

Formalin-fixed paraffin-embedded liver tissue was cut at 3 µm and stained with hematoxylin and eosin (H&E) according to standard procedures. Immuno-histochemical staining for T cells (CD3; #A0452; Dako) or macrophages/monocytes (Iba1; #019-19741, Wako Chemicals) were performed using the Ventana BenchMark XT autostainer (Roche Diagnostics, Tuscon, Arizona, USA) including heat mediated antigen retrieval. For detection of specific binding, the Ultra View Universal DAB Detection Kit (Ventana, Roche) was used which contains secondary antibodies, DAB stain and counter staining reagent for detection of nuclei. Representative pictures were taken with a Zeiss Axioscope 5 microscope and Axiocam 208 color camera.
